# Morphology in children’s books, and what it means for learning

**DOI:** 10.1038/s41539-025-00313-6

**Published:** 2025-05-05

**Authors:** Maria Korochkina, Kathleen Rastle

**Affiliations:** Department of Psychology, Royal Holloway, University of London, Egham, Surrey UK

**Keywords:** Human behaviour, Human behaviour

## Abstract

Breaking down complex words into smaller meaningful units (e.g., *unhappy* = *un-* + *happy*), known as morphemes, is vital for skilled reading as it allows readers to rapidly compute word meanings. There is agreement that children rely on reading experience to acquire morphological knowledge in English; however, the nature of this experience has remained unclear. We quantify the morphological information in 1200 popular children’s books and offer the first concrete description of how readers may learn affix morphemes through real-life text input. Our account considers the realities of morpheme presentation in natural language, such as the low number of distinct words in which affixes appear and the fact that their identification often requires specialised linguistic knowledge. This theory further accounts for the challenge posed by spelling patterns that may lead to incorrect morphological parsing. We conclude by exploring the implications of our findings for instructional programmes in morphology.

## Introduction

How do we make sense of words when we read? Consider the word *mistrustfulness*. You may have never encountered it before, yet you understand its meaning without effort. How does this happen? Research shows that we are able to understand complex words like *mistrustfulness* because we break them down into smaller, meaningful pieces: *mis-*, *-trust-*, *-ful*, and *-ness*^1^. These small units of meaning are called morphemes^2^. Somehow we know what each of these pieces means and how they combine to form a single word, even though we have not been taught this explicitly. The process of uncovering the meanings of words through a rapid analysis of their parts is known as morphological analysis, and it makes a fundamental contribution to our reading fluency and efficiency^1^. However, research also suggests that this ability to process words morphologically takes years of reading practice to develop^3^. In this paper, we investigate how we come to appreciate the meanings and functions of these smaller units within words. We use our findings to develop a theoretical account of how our reading experience shapes the morpheme learning process and discuss the implications for teaching practices and curriculum design.

Our article addresses these points with reference to derivational morphemes in English. Languages and writing systems vary substantially in how they communicate meaning through morphology [e.g., ref. ^4^]. Languages with rich morphology convey a substantial amount of grammatical information within individual words. For example, in most Slavic languages, verb forms change based on who is performing the action, how the action is performed, when it occurred, and whether it is carried out by an individual or a group^5^. In contrast, Swedish verb forms only convey information about when the action takes place. Languages also differ in the morphological structures they use. While Indo-European languages generally form words by stringing morphemes together sequentially, some languages favour certain constructions over others: for example, German makes much more extensive use of compounding than English (e.g., *Lieblings* ‘favourite’ + *Fußball* ‘football’ + *Mannschaft* ‘team’ → *Lieblingsfußballmannschaft* ‘favourite football team’)^6^. By contrast, Semitic languages do not use morpheme concatenation; instead, a word root is typically inserted into templates that indicate its function in a sentence. To illustrate, in Arabic, the root *k-t-b* is placed into various vowel patterns to create different words related to writing, such as $\underline{k}i\underline{t}\bar{a}\underline{b}$ ‘book’, $\underline{k}\bar{a}\underline{t}i\underline{b}$ ‘writer’, and $\underline{k}a\underline{t}a\underline{b}a$ ‘he wrote’^7^. Writing systems also vary in how they present words, and this influences the visibility of individual morphemes within those words. For example, Finnish and Tagalog both concatenate units of meaning to form words. However, in Finnish, the individual morphemes are typically easy to identify (e.g., *aamu* ‘morning’ + -$\underline{isin}\to aamu\underline{isin}$ ‘every morning’, *kesä* ‘summer’ + -$\underline{isin}\to$ *kesä*$\underline{isin}$ ‘every summer’), while in Tagalog, individual morphemes are often obscured due to nasal substitution and assimilation (e.g., $ma\underline{ng}$ ‘to do, to become’ + $\underline{p}ula$ ‘red’ $\to ma\underline{m}ula$ ‘to become reddish’, $ma\underline{ng}$ *+ bulag* ‘blind’ $\to ma\underline{m}bulag$ ‘to blind someone’)^8^. In comparison, in Arabic, the root morphemes (e.g., *k-t-b*) are salient in print because short vowels are typically not represented in everyday writing^9^.

Theoretical perspectives on reading propose that the process of acquisition involves encoding the statistical structure of a writing system in the minds of readers^4,10^. These theories imply that differences in morphological organisation across languages should have substantive consequences for what can be learned about morphology through print and how morphological information is analysed during reading. Thus, while many aspects of the role of morphology in reading that we discuss are relevant to other languages (and we highlight these explicitly), our findings regarding children’s experience of morphology through book reading and our conclusions about what they may learn through that experience are largely confined to English. Our work serves as an example of how a deep understanding of the information provided in print can provide new insights into core features of language acquisition and processing.

We begin by explaining what derivational morphology is and how morphological analysis contributes to skilled reading. We then turn to a discussion of the current understanding of how morphological knowledge is acquired, and how our study addresses the gaps in this research.

The morphemes of a language consist of free-standing morphemes, called stems, which provide a word’s lexical meaning (e.g., *-friend-* in *friendly* or *friends*), and morphemes, called affixes, that attach to stems to form new words (*-ly* in *friendly*) or word forms (*-s* in *friends*). The most common types of affixes are prefixes, which attach before the stem of the word (e.g., *un-*, *re-*, *mis-*), and suffixes, which attach after the stem (e.g., *-ly*, *-ness*, *-er*). Derivational morphology involves combining stem morphemes (e.g., *friend*) with affixes (e.g., *-ly*) to create new words (e.g., *friendly*). This process often involves a change of the lexical category of the stem: for instance, attaching the suffix *-ly* to a noun produces an adverb (*friend* + *-ly* → *friendly*), while adding the suffix *-ness* to an adverb results in a noun (*friendly* + *-ness* → *friendliness*). This is in contrast to inflectional morphology, where affixation results in a change of grammatical category, but the word’s core meaning remains the same (e.g., *-s* in *friends* denotes plurality and *-ed* in *walked* denotes past tense).

The combinatorial nature of derivation means that those words that share stem morphemes are usually related in meaning (e.g., *friendly*, *unfriendly*, *befriend*, *friendship*, *friendliness*). It also implies that affix morphemes contribute systematically to the meanings of the complex words in which they occur. For example, the prefix *un-* negates the stem to which it attaches, as in the words *unfriendly* (‘not friendly’), *uncommon* (‘not common’), *unafraid* (‘not afraid’), or *unaware* (‘not aware’). The suffix *-ify* forms verbs by adding the meaning ‘to make or cause to be [stem]’, as seen in words like *clarify* (‘make clear’), *diversify* (‘make diverse’), and *electrify* (‘cause to become electric’). The systematicity of this combinatorial system allows us to generalise — once we have learned that specific groups of letters have specific functions, we can use these spelling-meaning regularities as schemas with which new morphemic combinations can be interpreted (e.g., *boilable*, *yellowness*). Likewise, we can use the available inventory of stems and affixes to create new words on the fly (e.g., *brightify*, *friendlify*)^11–13^.

Words that consist of multiple inflectional or derivational morphemes, or compounds formed by combining stems (e.g., *snow* + *man* → *snowman*), are called *morphologically complex*. In this paper, we use this term to refer specifically to multimorphemic words formed through *derivational* affixation or compounding. In many languages, morphologically complex words make up a substantial percentage of the vocabulary. In English, for example, it is estimated that about 80% of words are compounds or consist of derivational affixes^14^, meaning that most unfamiliar words that English speakers encounter are new morphemic combinations^15,16^. Research shows that skilled readers of many languages take advantage of the morphological structure of their writing system to access meaning during reading [see ref. ^17^, for a review]. We discuss the place of morphology in reading in the next few paragraphs.

The core challenge of reading is to learn how visual symbols map to meanings. The way this challenge is met in different languages depends on how each language is represented in script^4^. In languages with alphabetic writing systems (e.g., English, Swedish, Armenian, Ukrainian), graphemes represent phonemes (e.g., in English, *d* → [d], *ng* → [ŋ]). In such languages, the relationship between spelling and sound is systematic, meaning that words that look similar also tend to sound similar (e.g., *tab*, *tap*, *tan*). In these languages, knowing the regularities between spelling and sound allows emerging readers to access the meanings of known printed words through their oral vocabulary and brings them to a level where they can begin to gain text experience^18–20^. It is therefore critical that children learning to read in alphabetic languages acquire the knowledge of the spelling-sound regularities in the first years of reading instruction.

Yet, the ability to decode visual symbols into sounds is not enough to support the extremely rapid word recognition and comprehension characteristic of skilled reading^18^. In contrast to novice readers, expert readers access the meanings of most words directly from their printed forms, without recourse to decoding [e.g., refs. ^21,22^]. Developing direct mappings between spellings and meanings enables readers to recognise words quickly and without conscious effort, allowing them to allocate more cognitive resources to comprehension [e.g., refs. ^23–26^]. But how are these direct links between spellings and meanings established? We cannot memorise the printed forms of all the words we know, especially since the relationship between spelling and meaning is often arbitrary: for example, *tab*, *tap*, and *tan* have similar spellings but are completely unrelated in meaning. This challenge might seem even more daunting when we consider that the average 20-year-old English reader is estimated to recognise around 71,400 distinct printed words^15^. In many alphabetic languages (e.g., English, Russian, German), an important part of the solution lies in a specific feature of their writing systems — that the mapping between spelling and meaning is underpinned by morphology [e.g., refs. ^1,10^].

The morphological organisation of the lexicon in these languages significantly reduces the number of distinct words readers need to learn by providing a way to interpret printed words through elements shared with other words. To illustrate, consider the English words *friend*, *friendly*, *friendship*, *befriend*, *boyfriend*, *friendliness*. If the reader understands how morphological regularities (e.g., that the suffixes *-ship* and *-ness* create nouns) are reflected in spelling, these different words can be seen as variations of the base word *friend*. Consequently, instead of learning each of these five words individually, the reader can use their knowledge of the word *friend* to infer the meanings of the other four. The significance of this becomes even more evident when we recall that a very large proportion of vocabulary in English and other alphabetic languages consists of multiple morphemes. Indeed, removing the inflectional variants from the estimated 71,400 words that the average English reader is expected to recognise^15^ would reduce the learning challenge to 42,000 words, and eliminating the derivational variants would further decrease this number to just 11,000 stems. This means that children would need to learn an average of two words per day, which is far more manageable than learning 71,400 printed words (equivalent to 12 new words per day). These figures illustrate why knowledge of derivational morphology is so powerful: it opens up substantial vocabulary to the reader. These figures also underscore what is at stake if children’s morpheme knowledge is suboptimal, highlighting the need to understand how morpheme learning occurs and whether current instructional practices could be optimised to facilitate it.

In addition to serving as an important heuristic for vocabulary growth, morphological knowledge helps us read faster. Research indicates that skilled readers leverage their knowledge of morphology not only to expand their vocabulary but also to accelerate the recognition of both familiar and unfamiliar printed words [see^1^, for a review]. For example, skilled readers are quicker at identifying printed words when their stems are high in frequency, as opposed to low [e.g., ref. ^27^], and when many other words share the same stem [e.g., refs. ^28–31^]. These findings imply that skilled readers are sensitive to morphological structure and analyse complex words (e.g., *kindness*) in terms of their constituent morphemes (e.g., *kind* + *-ness*) [see ref. ^17^], for a review].

Research further shows that skilled readers segment complex-looking words into morphemes in the earliest stages of word recognition, *before* accessing the meanings of these words. For instance, readers find it harder to determine that made-up morphemic combinations are not real words when these combinations consist of existing stems and affixes (e.g., *gasful* ← *gas* + *-ful*) compared to when they are formed from non-existent stems or affixes (e.g., *gasfil* ← *gas* + *-fil*, where *-fil* is not an English affix)^32,33^. Made-up words like *gasful* are segmented into smaller units because they *look* like real, morphologically structured words [e.g., refs. ^12,32,34^]. This segmentation occurs because it enables the rapid computation of meaning for both familiar and unfamiliar morphologically complex words^1^. A critical question is then how readers come to appreciate that some letter sequences form morphological units in written texts, and how this process is shaped by their experience with print. This research is only just emerging and, in the remainder of this section, we summarise the key insights to date and explain how the current study advances previous work.

Many of the points we made above regarding the role of morphology in vocabulary development and reading are applicable across languages. However, as we have argued earlier, languages differ in how they use morphology to convey meaning, and the acquisition of morphological knowledge in a particular language likely depends on the specifics of its writing system^4^. The focus of our work is on how morpheme knowledge is acquired in English, and therefore we limit the discussion below to literature that has examined this issue within this context.

Evidence abounds that the ability to use morphological information conveyed by the writing system to facilitate word recognition emerges gradually in reading acquisition and does not depend on the reader’s explicit knowledge of morphology. Research shows that, in English, knowledge of inflectional morphology develops faster than that of derivational morphology. Berko^35^’s seminal study demonstrated that children as young as four have already internalised some inflectional morphemes and can apply them to create new words. For instance, when presented with the sentences “Here is one wug. There are two of them. There are two …”, they are able to correctly produce $wug\underline{s}$. At this age, most children have not yet begun to learn to read, so their knowledge of inflectional morphology is thought to develop through experience of oral language [e.g., refs. ^36–39^]. By noticing phonological variation in familiar form-meaning pairs in speech (e.g., *one cat* – *two* $cat\underline{s}$), children discover linguistic rules, such as the plural being marked with [s], [z], or [iz]^37^. In contrast to her results regarding inflectional morphology, Berko^35^ found that young children’s understanding of derivational morphology was poor. For example, seven-year-olds failed to use the derivational suffix *-er* to produce agents (e.g., “This is a man who knows how to zib. What would you call a man whose job is to zib? He is a …”) or the suffix *-y* to describe a dog covered in *quirks* as $quirk\underline{y}$. Berko^35^ speculated that derivation may pose a challenge for young children due to limited exposure to derivational morphology in the spoken environment. Moreover, English derivation often involves phonological shifts (e.g., $div\underline{i}de$ *+ -ion* $\to div\underline{i}sion$) or changes in stress patterns (e.g., $m\underline{a}gic$ *+ -ian* $\to mag\underline{i}cian$), making the relationship between a derivation and its stem difficult to detect in speech. Thus, while young children demonstrate inflectional knowledge before learning to read, it is likely that the greater abundance and diversity of derivationally complex words in written relative to spoken language makes reading experience particularly important for the acquisition of derivational knowledge [e.g., refs. ^16,40^].

By age 9, children appear to have acquired basic knowledge of derivational morphemes (e.g., recognising the stems of complex words) and the ability to manipulate them explicitly [e.g., refs. ^41–44^]. By age 10, children are able to differentiate between morphologically structured made-up words like *quickify* and *gifter* and made-up words lacking apparent morphological structure (e.g., *quickilt*, *bulbow*)^45,46^. This body of research suggests that by the second half of primary school readers acquire sensitivity to certain distributional properties of stem morphemes, and have begun to develop the links between the conceptual representations of derivational affix morphemes and the letter strings used to represent these morphemes in print [cf. ref. ^47^]. Nonetheless, there is compelling evidence that this knowledge does not translate into the ability to rapidly segment printed words into morphemes until mid-to-late adolescence^3,46,48^. So, how do we acquire knowledge of derivational morphology, and why does it take several years to develop the stable morphological representations that support rapid morphological analysis in skilled readers?

Research on the mechanisms underlying affix learning is still in its early stages. However, emerging perspectives suggest that, in English, it is through their reading experience that children must discover the regularities between spelling and meaning. One reason for this is that pupils in English-speaking countries typically receive little explicit morphological instruction in school. For instance, the English National Curriculum specifies that simple inflectional (e.g., *-(e)s*, *-ing*, *-(e)d*) and derivational (e.g., *-er*) affixes should be taught in Year 1, when children are 5 to 6 years old^49^. It is recommended to introduce more complex derivational affixes progressively throughout the remaining years of primary school. Yet, this instruction typically focuses on accurate spelling, instructional time is limited, and research suggests that teacher knowledge of morphology is variable^50^.

In English, meaningful morphological information is also more salient in spelling than it is in the spoken language [e.g., refs. ^51,52^], and this is another reason why print experience may be particularly important for the acquisition of morpheme knowledge. To illustrate, the plural morpheme *-(e)s* can be pronounced like [s] (*cats*), [z] (*dogs*), or [iz] (*horses*), and the pronunciation of the past-tense ending *-(e)d* varies among [t] (*hoped*), [d] (*bobbed*), and [id] (*added*); yet, the spelling of these morphemes does not vary. The same pattern holds for derivational morphology: for example, the word-final sound sequence [ǝs] can be spelled in many different ways (e.g., $bon\underline{us}$, $atl\underline{as}$, $serv\underline{ice}$, $princ\underline{ess}$), but the spelling *-ous* is reserved to communicate adjective status (e.g., $hazard\underline{ous}$, $danger\underline{ous}$, $nerv\underline{ous}$)^53,54^. This ‘visibility’ of morphology in English orthography, compared to spoken language, suggests that exposure to print is likely a key factor in acquiring affix knowledge^1^. Research shows that readers are sensitive to statistical regularities in their linguistic environment [e.g., refs. ^55,56^]. Yet, currently we know little about the morphological information available in children’s written language environment and the challenges and opportunities it presents for learning the meanings and functions of derivational affixes. This paper sheds light on this issue by examining a large collection of popular children’s books and quantifying both the quantity and quality of the morphological information they provide. We draw on these insights to develop a concrete formulation of how the presentation of morphology in real-life reading may contribute to affix learning, and explore the practical implications of our findings.

What do we already know about learning morphemes without explicit instruction? Derivational affixes typically do not occur in isolation, and so their meanings and functions must be inferred from experience with the complex words in which they occur^47,57^. Laboratory experiments suggest that certain prerequisites are necessary for affix learning in the absence of instruction: one such prerequisite is that affixes alter the meanings of the stems to which they attach in highly predictable ways^58^. To illustrate, learning the prefix *un-* should be relatively straightforward as it virtually always means ‘not’ or ‘reverse action’, as in *unclean*, *unwise*, *undo*, *unscrew*, *unnoticed*. In contrast, learning the suffix *-ist* may be less straightforward because its function varies across different words. For instance, in *typist*, *racist*, *sadist*, and *heist*, the letter sequence *ist* appears in each word, but it serves different functions in each case. A *typist* is someone who types, but a *racist* is not someone who races. Both words are related in meaning to the stems they appear to contain (*type* and *race*), but the challenge lies in determining which meaning of the stems is relevant in each case (e.g., *race* as a category of human ancestry rather than *race* as a competition) and how the suffix *-ist* transforms this meaning. These examples alone illustrate that *-ist* links spellings to meanings with less consistency than *un-*.

The challenge of learning the function of *-ist* does not end there. Consider the word *sadist*: it might seem intuitive to segment it into *sad* and *-ist*, but a *sadist* is not someone who is sad, and the two words are unrelated in meaning. In fact, the word *sadist* is derived from the name of the Marquis de Sade, an 18th-century French nobleman. Finally, in the word *heist*, *ist* does not function meaningfully at all. Historically, *heist* is a local pronunciation of *hoist*, and is therefore unrelated to *he*, which might seem like a potential stem if we segment this word into constituents based on its spelling. Because the spellings of *sadist* and *heist* might lead us to analyse these words incorrectly (i.e., relating them to *sad* and *he*), they can be considered *pseudo-affixed*. The words *corner* and *brother* are further examples of pseudo-affixation as they are unrelated to their apparent orthographic stems, *corn* and *broth*. The challenge with such words is that they reduce the systematicity with which particular affixes are used, and in doing so, may disrupt learning. The observation that affixes vary in how consistently they convey a particular meaning highlights the graded nature of the regularity morphology provides in linking spellings to meanings and suggests that some affixes may be easier to learn than others.

Beyond the consistency of meaning, another key prerequisite for affix learning is that affixes appear with a large number of distinct stems^58^. Research suggests that, in this regard as well, there is considerable variation among the individual affixes. Dawson et al.^59^ analysed a corpus of 9582 short documents targeted at children aged 5–14 years. These documents were sampled from a broad range of contexts spanning fiction and nonfiction texts, curriculum materials, and text extracted from children’s websites. Dawson et al.^59^ cross-referenced the words in these documents against a database^60^ that provides information on the words’ morphological structure based on their origin. They then analysed the distribution of different suffixes across the documents in their corpus. Dawson and colleagues observed that, in texts for the younger primary school children, the most common suffixes were those that are also the most common in spoken language (e.g., *-ly*, *-y*, *-er*, *-ful*). By contrast, texts for children aged 13–14 years that they analysed were dominated by suffixes of Latinate origin which are used to create deverbal nouns (e.g., *celebrate* + *-ion* → *celebration*, *educate* + *-ion* → *education*, *disturb* + *-ance* → *disturbance*, *secure* + *-ity* → *security*). These suffixes often induce changes to the stress and phonology patterns of the stems to which they attach (e.g., $\underline{a}tom$ *+ -ic* $\to at\underline{o}mic$, $prod\underline{u}ce$ *+ -ion* $\to prod\underline{u}ction$), making them difficult to recognise in the spoken forms of the words. Finally, Dawson et al.^59^ reported that the proportion of suffixed words increased in line with the target age of the text. Interestingly, fiction books aimed at children aged 7 and older were found to contain a higher proportion of distinct suffixed words than nonfiction texts for the same age group^59^.

The study by Dawson et al.^59^ is the first large-scale investigation of morphological complexity in children’s reading materials. This initial investigation is important because it highlights that suffixes vary in how well they are represented in children’s books and that the meanings of some suffixes may become embedded in the reading system earlier in development than others. Yet, this study does not address a critical aspect of how morphology is reflected in writing: that words that *appear* complex may not necessarily contain derivational affixes, while words identified as multimorphemic in a dictionary may not always *look* complex. Recall the issue of pseudo-affixation discussed earlier: words like *corner* and *heist* look like they contain the suffixes *-er* and *-ist*, and this may have consequences for learning. The word *subside* presents a similar challenge: based on its spelling, it would be intuitive to segment this word into the prefix *sub-* and the English word *side*. Yet, *subside* is derived from the Latin verb *sidere* ‘to settle’ and is unrelated to *side*, so this orthographic analysis would be misleading and will likely harm the reader’s learning of the prefix *sub-*.

Likewise, etymological knowledge is often required to identify affixes within genuinely complex words. Consider the words *subconscious*, *subheading*, and *suboptimal*. We can easily detect the prefix *sub-* in these words because the stems *conscious*, *heading*, and *optimal* are meaningful and occur on their own. Now consider the words *subject*, *submit*, and *subjugate*: if we remove the prefix *sub-*, we are left with *ject*, *mit*, and *jugate*. These stems cannot stand on their own as words and do not have any meaning in modern English. They originate from the Latin words *jacere* ‘to throw’, *mittere* ‘to send’, and *jugum* ‘yoke’; however, without consulting a dictionary, the average reader will not be able to recognise them. These stems are called *bound stems*, and they must be combined with at least one affix to form meaningful words. Children’s etymological knowledge is limited at best, so it is unlikely that experience with words like *submit* would help them understand the function of the prefix *sub-*. Importantly, such words are not exceptions: linguistic research suggests that, in English, there may be more complex words with bound stems than there are complex words with free-standing stems^61^. These examples clearly demonstrate why dictionary-based etymological counts on their own are not enough to judge the exposure to morphology that children receive through independent reading and whether this exposure is sufficient to support morpheme learning.

The analytical approach used in our study is based on the premise that the idiosyncrasies in the ease with which complex words can be parsed into morphemic constituents (e.g., *subconscious* vs. *subject*) and the extent of pseudo-affixation (e.g., *corner*, *sadist*) will have a bearing on children’s learning of morphemes. Dawson et al.^59^ defined complex words based on etymology and focused solely on suffixes; additionally, some subcorpora in their study included only a small number of texts (e.g., 14 in the nonfiction sample for the 5–7 age group, with only 1864 distinct words), and the largest subcorpus contained just 18,487 distinct words. Our study advances beyond this earlier investigation by using a significantly larger corpus of children’s books and including both prefixes and suffixes in our analysis. Critically, we also evaluate how easily different affixes can be detected within words based on the information provided in spelling alone, without recourse to sophisticated etymological knowledge. We build on our findings to formulate a concrete theoretical account of how children may learn about morphology through real-life reading experience. Our analytical approach is detailed in the Methods section at the end of the article. In the following section, we report our key findings, each accompanied by a brief discussion, before exploring the broader implications of our results for theories of morphology acquisition and the teaching of morphology in the final section.

## Results and discussion

To understand the nature of morphological information that children are exposed to when they read for pleasure in English, we drew on the Children and Young People’s Books Lexicon (CYP-LEX)^62^. CYP-LEX is a database of 105,694 distinct words derived from an analysis of 1200 books popular with British children, including 400 books suitable for each of three age bands (7–9 years, 10–12 years, and 13–16 years). Our starting point followed the approach of Dawson et al.^59^ in establishing the morphological structure of words in CYP-LEX on the basis of etymological information available in the MorphoLex database^60^. However, as we have argued in the previous section, children’s etymological knowledge is typically limited, and therefore judging their exposure to morphological information on the basis of etymological counts alone can be misleading. We addressed this issue by means of a second analysis, where we quantified how easily affixes can be detected by a reader on orthographic grounds alone. In the remainder of this section, we report the outcomes of all analyses along with a brief discussion of each finding, and we refer the reader to the Methods section for further details on how the analyses were conducted.

### Books contain many morphologically complex words

Table 1 reports the number of distinct words in each of the CYP-LEX age bands that were available for the analysis using the MorphoLex database, along with the number and percentage of those words that are morphologically complex.

**Table 1:** Number (*N*) of distinct words in each CYP-LEX age band for which information is available in MorphoLex, along with the number and percentage (%) of morphologically complex words among them

	The CYP-LEX age band		
	7–9	10–12	13+
*N* distinct words with entries in MorphoLex	39,151	47,365	54,559
*N* (%) morphologically complex words	17,634 (45%)	22,564 (48%)	27,555 (51%)

These data make clear that morphologically complex words comprise about half of the distinct words in children’s books. Table 1 further demonstrates that the proportion of complex words increases in line with book target age, suggesting that exposure to morphology intensifies as books become more advanced. Indeed, our analysis shows that most words in books for older children that do not appear in books for younger children consist of multiple morphemes: 61% (*N* = 6276) of the words that occur in the 10–12 but not 7–9 age band are morphologically complex, and 68% (*N* = 6317) of the words that occur in the 13+ but not 10–12 age band are morphologically complex.

The fact that so many words in children’s books are multimorphemic highlights the complexity of vocabulary in popular books from the earliest years of independent reading. Moreover, our findings also lend support for the idea that, as children age, most new words that they encounter through reading are new combinations of meaningful units (morphemes) that they already know [e.g., ref. ^16^]. Our data thus emphasise the importance of morphological knowledge: children will struggle to read for meaning if they do not understand how morphology governs the relationship between spelling and meaning.

### Books are a unique source of morphological information

To better understand whether and how books differ from spoken language regarding the amount of morphological information they provide, we studied the structure of words used in children’s books versus the structure of words used on British television. This analysis relied on the lexical statistics provided in SUBTLEX-UK, a publicly available database of every word used in programmes broadcast on nine BBC channels over three years^63^. One of the nine channels included in SUBTLEX-UK is CBBC which targets children aged 6–12 years. This age range overlaps with two of the CYP-LEX age bands, 7–9 and 10–12. Therefore, we compared the morphological information in the CYP-LEX age bands with that in CBBC (reflecting exposure in children’s television programmes) and the entire SUBTLEX-UK (reflecting exposure in programmes targeted at audiences of all ages).

This analysis revealed that there are a substantial number of words in children’s books that do not occur in either the CBBC or the SUBTLEX-UK databases (see Table 2). Television language is often used as a proxy for spoken language [e.g., refs. ^64–66^]. Thus, these numbers indicate that children may encounter many words in books that are not present in their spoken language environment. Moreover, this analysis revealed that the majority of these ‘new’ words are morphologically complex.

**Table 2:** Number (*N*) of distinct words in each CYP-LEX age band for which information is available in MorphoLex that do not occur in the CBBC or SUBTLEX-UK databases, along with the number and percentage (%) of morphologically complex words among them

	The CYP-LEX age band		
	7–9	10–12	13+
*N* distinct words missing from CBBC	8280	14,050	20,105
*N* (%) complex words among those missing from CBBC	4924 (59%)	8562 (61%)	12,894 (64%)
*N* distinct words missing from SUBTLEX-UK	1211	2450	4602
*N* (%) complex words among those missing from SUBTLEX-UK	888 (73%)	1796 (73%)	3514 (76%)

Most of these morphologically complex words have low frequency (see next section); however, our analyses showed that the percentage of complex words frequently used in books (raw frequency of 50 and higher) but not encountered on CBBC increases in line with book target age (1% in the 7–9 age band, 2% in the 10–12 age band, 5% in the 13+ age band). This property of children’s books implies that they provide a richer source of information for learning about morphology than spoken language.

### Few complex words are used repeatedly in books

We have said above that about half of the words in each age band for which information was available in MorphoLex are morphologically complex. We have also shown that, for each age band, multimorphemic words constitute a large proportion of words missing on television or in books for younger children. Critically, however, our next analysis suggests that very few of the morphologically complex words are used *repeatedly* in books.

Our analyses demonstrated that multimorphemic words are used much less frequently in children’s books than monomorphemic words (see Table 3). For instance, in the 7–9 age band, half of the distinct complex words are used no more than 5 times across the 400 books included in this age band, and only 8% of complex words are repeated at least 100 times. In contrast, less than a third of the distinct monomorphemic words in this age band have a frequency of 5 or less, and 23% of monomorphemic words occur 100 times or more. The data in Table 3 indicate that, as books become more advanced, the percentage of frequent complex words increases. Nonetheless, the figures suggest that, although there are many morphologically complex words in children’s books, most of these words are used rarely.

**Table 3:** Frequency of multimorphemic and monomorphemic words in the CYP-LEX corpus

		The CYP-LEX age band		
		7–9	10–12	13+
Multimorphemic words	*N* distinct words	17,624	22,564	27,555
	Encountered ≤ 5 times	50%	42%	35%
	Encountered ≥ 100 times	8%	11%	15%
Monomorphemic words	*N* distinct words	21,517	24,801	27,004
	Encountered ≤ 5 times	30%	24%	18%
	Encountered ≥ 100 times	23%	29%	36%

For each CYP-LEX age band, the table reports the number (*N*) of distinct multi- and monomorphemic words, and the percentage of words among them that are used either 5 times or less, or 100 times or more, across the 400 books in this age band.

Our analyses also revealed that morphologically complex words are poorly distributed across the individual books. In the 7–9 age band, only 1% of multimorphemic words are encountered in at least half (≥ 200) of the books, and only 3% are used in at least one quarter (≥ 100) of the books. Like frequency, the distribution of morphologically complex words across the individual books also tends to improve as a function of book target age: 5% and 7% of complex words in the 10–12 and 13+ band, respectively, occur in 100 books or more. However, these numbers are still low compared to the percentage of monomorphemic words used in 100 books or more: 13% in the 7–9 age band, 17% in the 10–12 age band, and 21% in the 13+ age band.

Together, these findings demonstrate that children are *highly likely* to encounter a morphologically complex word when they read for pleasure; however, they are *highly unlikely* to ever see this particular complex word again. As a consequence, it will be difficult for children to learn to recognise morphologically complex words by sight, meaning that their reading fluency and efficiency will be determined by the ability to break multimorphemic words apart.

### Greater exposure to suffixed than to prefixed words in children’s books

Having established that morphologically complex words constitute a large proportion of words in children’s books but are low in frequency and poorly distributed across the individual books, we turned to an analysis of the structure of the multimorphemic words used in children’s literature.

**Fig. 1:**
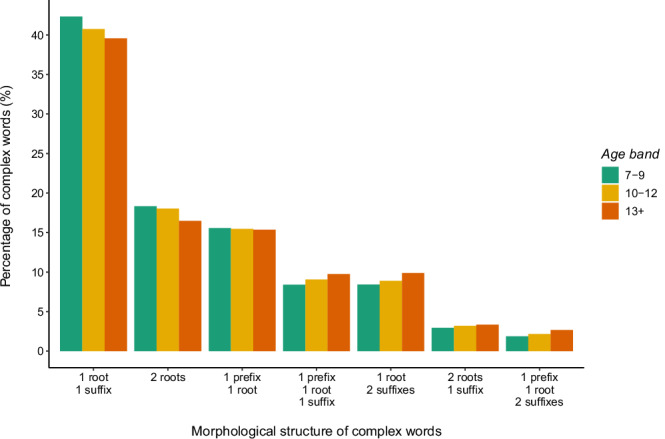
The most common types of morphological structure observed in CYP-LEX. The x-axis represents the different structure types, and the y-axis shows the percentage (%) of distinct morphologically complex words for each structure type. The colours indicate the three age bands: green for ages 7–9, yellow for ages 10–12, and orange for ages 13–16.

Figure 1 illustrates the most common types of morphological structure observed in books. Most multimorphemic words include at least one affix, and about 17% of the complex words are unaffixed compounds. It is interesting that the figures do not vary much across the three age bands: this suggests that the distribution of different morphological structures in children’s literature remains largely invariant as children transition to more advanced texts.

Figure 1 also shows that suffixed words dominate over prefixed words in popular children’s books: in each age band, more than two thirds of complex words include at least one suffix (66%, 66%, and 67% in the 7–9, 10–12, and 13+ age bands, respectively). In contrast, prefixes are encountered in less than one third of multimorphemic words (27%, 28%, and 29% in the 7–9, 10–12, and 13+ age bands, respectively). Moreover, most of the suffixed words do not contain any prefixes (83% in the 7–9 age band, 82% in the 10–12 age band, and 80% in the 13+ age band), while, among the prefixed words, almost half (41%, 43%, 46% in the 7–9, 10–12, and 13+ age bands, respectively) also include at least one suffix. These numbers indicate that, in each age band, exposure to suffixes is far greater than exposure to prefixes.

The prefixed words are also repeated less often than the suffixed words. In the 7–9 age band, 10% of the prefixed words are used 50 times or more, compared to 14% for the suffixed words, and this relationship is also maintained in books for older children (15% vs. 19% in the 10–12 age band, 23% vs. 25% in the 13+ age band). As books become more advanced, the difference in frequency between the two types of affixed words appears to become smaller, suggesting that a larger number of prefixed words are used frequently in books for older children than in books for younger children. Nevertheless, among those morphologically complex words that are encountered in at least half (≥ 200) or in at least one quarter (≥ 100) of the books, the majority (64% and 67% in the 7–9 age band, 67% and 67% in the 10–12 age band, and 66% and 65% in the 13+ age band, respectively) include suffixes but not prefixes. These findings underscore the importance of reading widely: only by exposing themselves to many texts will children be able to experience different affixes with different stems. And yet, even for those who do read widely, exposure to the prefixed words is likely to be very limited, particularly in books for primary school children.

### Few affixes are used with many distinct stems

**Fig. 2:**
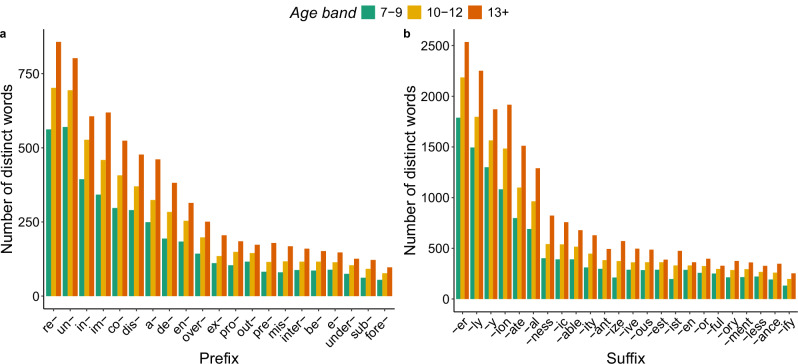
Type frequency of the most common affixes across the three age bands. Panel (**a**) displays prefixes, and panel (**b**) shows suffixes. Type frequency refers to the number of distinct words that contain a given affix. Notably, the values on the y-axis in panels (**a**) and (**b**) differ substantially, reflecting the fact that suffixes are used in many more distinct words than prefixes.

The focus of the analyses reported above was with the affixed *words*, and we now turn to discussing the individual *affixes* encountered in these words. Figure 2 illustrates, for each age band, the type frequency (i.e., the number of distinct words in which an affix is encountered) of the 48 most common affixes, and the exact type frequency and token frequency (i.e., total number of words in which an affix is encountered) values for all affixes are reported in Supplementary Data 1.

It is immediately apparent from Fig. 2 that, overall, suffixes occur with a much larger number of distinct stems than prefixes do: there are ten suffixes (*-er*, *-ly*, *-y*, *-ion*, *-ate*, *-al*, *-ness*, *-able*, *-ic*, *-ity*) but only four prefixes (*un-*, *re-*, *in-*, *im-*) that are encountered in at least 300 distinct words in each age band. It is striking that even in the 13+ books, the type frequency of 65% of the prefixes is lower than 300 words, suggesting that children’s experience with these prefixes may be too impoverished to enable learning^58^. Overall, the type frequency of each affix increases in line with book target age; however, it is important to realise that both the total number of words (book length) and the number of distinct words also increase as books become more advanced. Thus, these data clearly show that, for each suffix, exposure to multiple distinct words is limited before the 13+ texts, while, for the prefixes, it remains limited even in these more advanced books.

### Few affixed words are used repeatedly across books

**Fig. 3:**
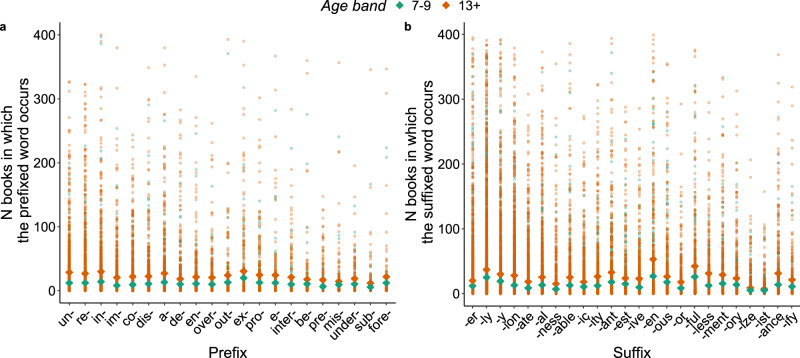
The number of books in which words containing the most common affixes occur. Panel (**a**) displays prefixes, and panel (**b**) shows suffixes. Data for the 7–9 age band are represented in green, and the data for the 13+ age band are shown in orange. Data for the 10–12 age band are not shown for ease of figure interpretability. Each coloured circle corresponds to a distinct word with a given affix. The diamond-shaped points at the bottom of each panel represent the mean number of books in which all words with a given affix occur.

Our analysis further shows that the affixes are sparsely represented across the individual books. For each affix, Fig. 3 reports the number of books in the 7–9 and in the 13+ age bands in which each word containing this affix is encountered. This figure demonstrates that the average affixed word does not occur in many books and that the suffixes are much better represented across the books than the prefixes. For each affix, the number of words that occur in multiple books increases in line with book target age, suggesting that the affixes tend to become better distributed across the individual books as children progress to books aimed at older readers. Nonetheless, it is evident from Fig. 3 that only a few affixes have reasonable representation across books before the 13+ age band.

### A different approach: morphemes defined orthographically

So far, we have reported statistics derived from an analysis in which morphemes were defined on the basis of the words’ etymology. However, we argued in the beginning of this section that this approach can be misleading because it does not take into account the ease with which individual morphemes can be detected by readers with limited knowledge of etymology. We have illustrated this point in the Introduction using the words *submit*, *subjugate*, and *subject*; further examples of morphologically complex words with bound stems include words like *depend*, *consume*, and *inflict*. These words are built by adding the prefixes *de-*, *co-*, and *in-* to Latin verbs *pendere* ‘to hang’, *sumere* ‘to take up’, and *fligere* ‘to strike’. Critically, because *pend*, *sume*, and *flict* are not words in English, it is unlikely that children would analyse words like *depend*, *consume*, or *inflict* in terms of their morphemes.

One could argue that understanding the exact meanings of bound stems may not be necessary for morphological segmentation. For example, a child might notice that certain letter sequences appear in multiple complex words (e.g., *-clude-* in *include*, *exclude*, *preclude*) alongside other letter sequences (e.g., *in-*, *ex-*, *pre-*). They could then use this information to break complex words into components (e.g., *in-* and *-clude-* in *include*). Yet, the segmentation derived in this way is unlikely to aid in learning the *meanings* of the affixes. While a child might identify *in-* in *include*, learning the meaning of *in-* from this word is probably less intuitive than from words like *inconsistent* or *incomplete*, where *consistent* and *complete* function as standalone words in English. This, in turn, could impact a child’s ability to infer the meanings of rare or unfamiliar words that contain *in-*. Therefore, while some degree of segmentation may be possible for complex words with bound stems, its usefulness regarding affix learning is likely to be limited.

Parsing problems may also arise when encountering pseudo-affixed words like *corner*, *brother*, and *forty*. Although each of these words can be segmented into meaningful units (*corn* + *-er*, *broth* + *-er*, *fort* + *-y*), the result of this orthographic segmentation would be misleading: the words *corner* and *brother* appear complex but, in reality, do not contain the suffix *-er* and are unrelated to the words *corn* and *broth*. Likewise, the word *forty* is not related to the word *fort*; it originates from the Old English *fēowertig*. Because words like these look morphologically complex but cannot be traced back to their apparent orthographic ‘stems’, they can be thought of as morphological *false alarms*. Thus, in our next analysis, we sought to assess how many of the truly complex words can be detected by a reader operating on the basis of orthography, and, conversely, how many words are likely to be parsed incorrectly based on their spelling (*forty* parsed as being related to *fort*; *corner* parsed as being related to *corn*).

We developed an algorithm that tested whether whole words in the CYP-LEX database could be segmented into morphemes based on orthographic information. This algorithm relied on a pattern matching technique called Regular Expressions, or RegEx, which checks whether a given sequence of characters is present in a given letter string. To illustrate, the word *teacher* can be segmented into the English word *teach* and the suffix *-er*. This type of segmentation can also be applied to the pseudo-suffixed word *corner* (*corn* + *-er*) such that this word would also count as an instance of the *-er* suffix on orthographic grounds. In contrast, the word *infer* also contains the orthographic pattern *er*; however, in this case, the removal of *er* will result in *inf*, which is not an English word. Thus, the word *infer* would not add to a reader’s experience of the suffix *-er*. The Methods section provides detailed information on how this algorithm was implemented, and the output is available in Supplementary Data 2–4. It is important to recognise that the numbers reported in the appendices and below are estimates and may vary depending on the specifics of the algorithm used to generate them. For this reason, our interpretation relies not on the exact figures but on the overall perspective they provide regarding exposure to morphology in children’s books. In the remainder of this section, we report the outcomes of the morpheme detectability analysis and their possible implications for morpheme learning.

### Few affixes can be detected without etymological knowledge

**Fig. 4:**
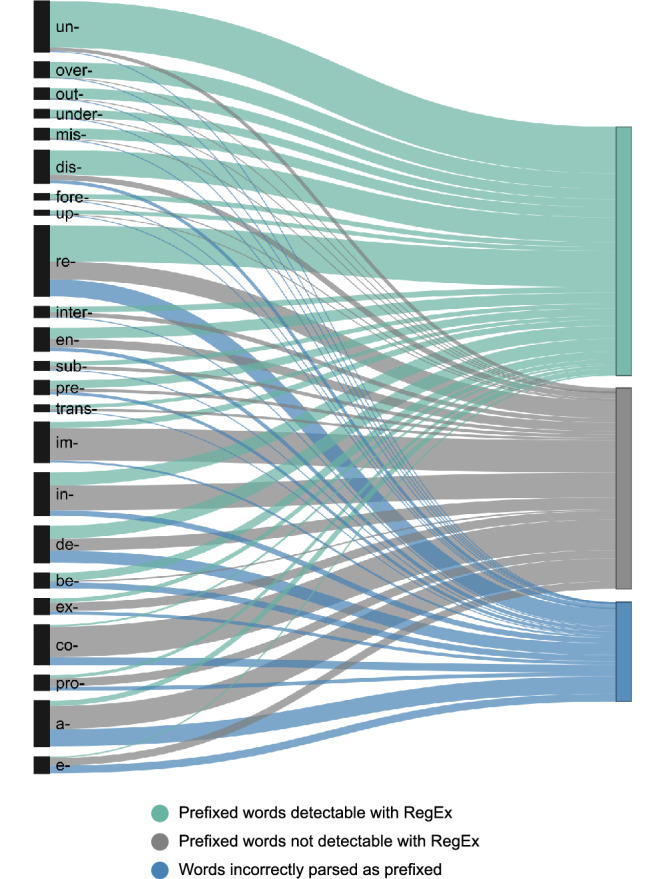
Orthographic detectability of the 23 most common prefixes in the CYP-LEX corpus across all age bands. Each prefix is represented by a black vertical bar, with its height indicating the number of distinct words identified as containing that prefix based on their spelling. The colours denote the detectability status of the words: green represents genuinely complex words detected by the RegEx algorithm, grey represents genuinely complex words not detected by the algorithm, and blue represents pseudo-prefixed words.

**Fig. 5:**
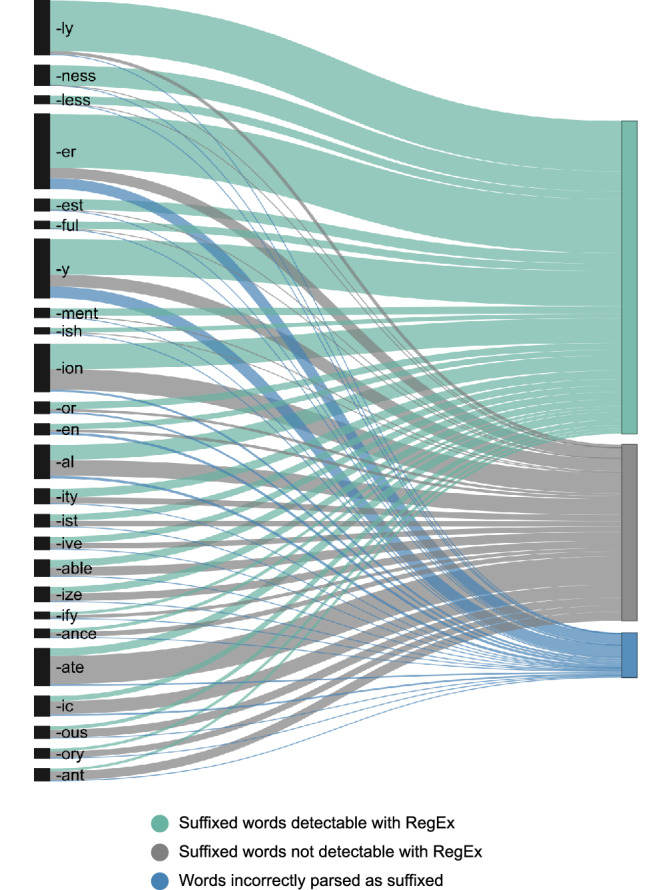
Orthographic detectability of the 25 most common suffixes in the CYP-LEX corpus across all age bands. Each suffix is represented by a black vertical bar, with its height indicating the number of distinct words identified as containing that suffix based on their spelling. The colours denote the detectability status of the words: green represents genuinely complex words detected by the RegEx algorithm, grey represents genuinely complex words not detected by the algorithm, and blue represents pseudo-prefixed words.

Figures 4 and 5 visualise the numerical data available in Supplementary Data 4 for the entire CYP-LEX database (i.e., not grouped by age band but extracted from the entire collection of the 1200 books). In these figures, each prefix (Fig. 4) and each suffix (Fig. 5) is represented by a black vertical bar, and the height of the bar corresponds to the number of distinct words with this prefix or suffix (including both truly complex and pseudo-affixed words) available across the 1200 books. The three colours represent the detectability status of each word: morphologically complex words containing a given affix that were detected by the RegEx algorithm appear in green; morphologically complex words containing a given affix that were not detected by the algorithm are shown in grey; and pseudo-affixed words (i.e., potential false alarms) are depicted in blue.

The aim of the RegEx analysis was to simulate a reader who has limited knowledge of etymology. Figures 4 and 5 reveal that this reader will be unable to detect the derivational morphemes in approximately half of all genuinely prefixed words and about a third of all genuinely suffixed words. The number of ‘hits’ is low because a large proportion of morphologically complex words have bound stems and thus cannot be segmented into smaller units without the knowledge of their etymology (e.g., *agony*, *pessimist*, *institute*, *contain*, *depend*). Some words with bound stems pose an even more challenging parsing problem due to complex orthographic alterations. For example, the word *sustain* was formed by adding the prefix *sub-* to the Latin stem *tenere* ‘to hold’, creating *sustinere*, which later entered English as *sustain* via the French *soustenir*. While words like *subject* and *submit* at least retain the orthographic pattern *sub*, the word *sustain* lacks this pattern entirely, meaning that it cannot be used to inform the meaning of the prefix *sub-*. Further examples of complex words in which the prefix is hidden in modern spelling include words like *entertain* (*inter-* + *tenere* → *entretenir* → *entertain*), *effect* (*ex-* + *facere* ‘to do, to make’ → *efficere* ‘to accomplish’ → *effectus* → *effect*), and *corrupt* (*co-* + *rumpere* ‘to break’ → *corrumpere* ‘to mar, to destroy’ → *corruptus* → *corrupt*). The finding that there are many words in children’s books whose morphological status is obscured is striking: it indicates that the number of distinct words that a child will be able to use to infer the meanings and the functions of derivational morphemes is much lower than that expected based on the MorphoLex type frequency data. We reported earlier in this section that most affixes occur with only a small number of distinct stems, meaning that children’s experience with most affixes is likely to be rather impoverished. The RegEx data demonstrate that the estimates based on these etymological counts are too optimistic and that the opportunity for affix learning through reading is in fact much more limited.

While the *overall* detectability of the derivational affixes is low in children’s books, Figs. 4 and 5 clearly show that there is substantial variation across the individual affixes regarding the ease with which they can be detected. For instance, our algorithm was able to detect over 93% of all complex words containing the prefix *un-* and the suffix *-ly*; however, it could identify only 6% of words with the prefix *co-* and no more than 22% the suffix *-ate*. This is because many words with these affixes contain stems that do not occur in isolation in modern English. Examples include words like *coherent* or *coincide* — *herent* and *incide* are not valid English words. The situation is further complicated by the fact that the prefix *co-* has several variant forms (*com-*, *con-*, *cor-*) such that, in MorphoLex, words like *compose*, *contain*, *convert*, *concept*, *conflate*, and *corrupt* are also tagged as words with the prefix *co-*. Similarly, many words with the suffix *-ate* are words with bound stems: consider, for instance, the words *appreciate*, *articulate*, *generate*, *intimidate*, *eliminate*, *disseminate*, *assimilate*. These words are used relatively frequently; however, their stems, many of which are themselves multimorphemic (e.g., *a(p)-preci-*, *-in-timid-*, *-e-limin-*, *-dis-semin-*, *-a(s)-simil-*) are not observed in isolation in English. It is interesting that the detectability of an affix is not necessarily associated with its frequency: suffixes *-y*, *-er*, and *-ly* are the top three suffixes regarding the number of distinct words they appear in, yet, the detectability of *-y* and *-er* is substantially lower than that of *-ly*. In contrast, there are few distinct words with the prefix *under-*, and all but two of these words were detected by the RegEx algorithm.

It is further evident from Figs. 4 and 5 that, on average, pseudo-affixation is not a very common phenomenon in children’s books: no more than 3% of unprefixed and no more than 5% of unsuffixed words (based on etymological information) were identified as containing prefixes or suffixes. This result is in line with previous work suggesting that pseudo-affixed words are exceptional in English orthography [e.g., ref. ^54^]. However, although false alarms are relatively infrequent overall, certain affixes appear to be significantly more affected by this issue than others. For instance, there were very few false alarms for *un-* and *-ly*; however, for *co-*, the number of false alarms was about 4 times the number of hits. Examples include words like *comedian*, which can be parsed as *co-* + *median*, ‘the middle point in a dataset’, or *copiously*, which can be segmented into the prefix *co-* and the stem *piously*, from *pious*, ‘devoutly religious’.

Together, these results indicate that only a small set of affixes are easily detectable and occur in many different words, while the majority of affixes are both difficult to detect and occur with a small number of distinct stems. One crucial implication of this finding is that, beyond a handful of affixes, morpheme knowledge may be difficult to acquire from text. It is important to remember that our analysis relied on 400 books in each age band; clearly, no child can be expected to read that many. Moreover, recent data suggest that less than 30% of children and young people read daily in their free time^67^, suggesting that most children’s experience of morphology will be much more limited than that described here. These considerations suggest that a discussion about explicit morphology instruction may be in order, and we turn to this issue in the following section.

## General discussion

The aim of this study was to advance theoretical understanding of how children’s experience with print contributes to their acquisition of morphological knowledge in English. We used a corpus of over 100,000 distinct words that British children aged 7–16 encounter when they read for pleasure to capture and quantify the morphological information available in popular children’s books. Our analysis revealed that nearly half of the distinct words in children’s books are morphologically complex and that many of these complex words are never encountered in BBC television programmes targeted at children of the same age or at adults. Previous research indicates that picture books contain more morphologically complex words than child-directed speech^68^, and our findings demonstrate that this trend persists in the books that children read independently. It is important to recognise that books included in our corpus were selected based on their popularity rather than their literary or linguistic merits. Thus, our results suggest that even popular, non-curated, books provide a richer source of information on derivational morphology than spoken language. We also found that thousands of words in books for older readers, which are not found in books for younger children, consist of multiple morphemes [see also ref. ^62^]. This finding further underscores the opportunity that popular books provide for children’s exposure to morphology, even as children progress to more advanced reading materials.

However, our analysis also revealed the significant learning challenge children face from the earliest years of independent reading. Although morphologically complex words comprise a large proportion of book vocabulary in each age band, very few of these words are used repeatedly within and across books. In other words, our data show that children are *highly likely* to encounter a multimorphemic word when they read but *highly unlikely* to ever see this particular word again, even if they read widely. This distributional characteristic — that a small number of linguistic units are highly frequent while the majority are low in frequency — is not unique to morphologically complex words or written texts. Rather, it is observed across all words in both spoken and written language [see, e.g., ref. ^69^, for a review]. For example, a recurring pattern observed in numerous language corpora is that the most common word appears approximately twice as often as the second most common, three times as often as the third, and so on^70^. When applied to the learning of morphology through print, this property of book vocabulary means that it will be difficult for children to learn to recognise many complex words by sight — a process known as orthographic learning^21,22^. As a result, their reading fluency and efficiency will depend on their ability to break complex words into their component parts. But how do readers acquire the knowledge necessary to do this?

Children encounter basic morphological structures (e.g., plural forms such as *dogs*, or past tense forms such as *walked*) through spoken language and early reading experiences [e.g., refs. ^71,72^]. They are taught to recognise and manipulate some derivational morphemes as part of formal literacy instruction and gradually learn to appreciate that many words are built from smaller parts (e.g., *un-* + *happy* → *unhappy*). However, there is typically little time for explicit morphological instruction in the school curriculum, and teacher knowledge of morphology is often sparse [e.g., ref. ^50^]. This leaves pupils needing to rely on their linguistic experience to discover what information different morphemes communicate. The structure of the English writing system is thought to be helpful in this endeavour because many morphological regularities are more prominent in written than in spoken language [e.g., refs. ^10,53,54^]. Theories of skilled reading therefore propose that the acquisition of morphological knowledge is governed by children’s experience with print^1^.

Research unequivocally shows that, regardless of what is being learned, the ability to generalise requires exposure to highly variable input [see ref. ^73^, for a comprehensive review]. If variability in our environment is low, we may be able to learn isolated events quickly, but we are unlikely to learn to generalise those experiences to new situations. In the case of morpheme learning in the absence of explicit instruction, this principle implies that learning the meanings of affixes requires that they occur in combination with many different stems^58^. Another critical prerequisite for affix learning is that the reader is able to identify the affixes within the complex words in which they occur. For example, words like *agony*, *amnesia*, *anaemia*, and *atom* are of Greek origin and historically contain the prefix *a-* (e.g., *á- +* $mimn\acute{\bar{e}}sk\bar{o}$ ‘to remind, to remember’ → *amnēsía* ‘forgetfulness’ → *amnesia*; *á-* + *témnein* ‘to cut’ → *átomos* ‘indivisible’ → *atom*). However, in modern English, these words have bound stems that cannot stand alone as words (e.g., *mnesia* and *tom* are not independent words), making it unlikely that readers would be able to segment them into smaller meaningful parts or use their experience with such words to learn about affix *meanings*. In some cases, this issue is further complicated by orthographic alterations that obscure the affix morpheme. For example, the word *sustain* does not include the letter sequence *sub*. Historically, however, *sustain* is derived from adding the prefix *sub-* to the Latin stem *tenere*, making it classified as a word with the prefix *sub-* in the dictionary. The presence of bound stems combined with orthographic changes makes it unlikely that readers would use their experience with these words to infer the functions of *a-* and *sub-* unless they are aware of the words’ etymology. The issue of morpheme detectability thus emphasises the need to distinguish between what is classified as morphologically complex in a dictionary and what a child reader is likely to perceive as complex. However, this distinction has been overlooked in previous studies of morphological complexity in children’s books in English [e.g., refs. ^44,59,68^].

The work presented here offers the first *concrete* description of how children may learn about morphology through the *graded* input available in real-life reading. This description relies on a thorough examination of the frequency with which the most common affixes are used with different stems (i.e., their type frequency), the extent of their usage with bound stems, and instances involving orthographic alterations. We found that the type frequency of each affix increases as the books become more advanced. However, our analysis also revealed that, for most suffixes, exposure to multiple types can be considered adequate only in books aimed at children aged 13 and older, whereas for the prefixes, exposure remains limited across all age bands. Affixes associated with greater variability in the stems they combine with include suffixes *-er*, *-ly*, *-y*, *-ion*, *-ate*, *-al*, *-ness*, *-able*, *-ic*, and *-ity* and prefixes *un-*, *re-*, *in-*, and *im-*, and examples of affixes that are used in only a few distinct words are suffixes *-ify*, *-ory*, and *-ance/-ence*, and prefixes *fore-*, *e-*, and *be-*. It is important to note that these data are based on 400 books per age band, which is more than any child is expected to read. Thus, these count data alone already indicate that children’s exposure to most affixes is likely to be insufficient to enable effective learning.

Yet, the full extent of the problem only becomes apparent when we examine these data alongside the results of our morpheme detectability analysis. This analysis shows that derivational affixes will be difficult to detect in approximately half of all prefixed words and a third of all suffixed words, significantly limiting the experience with complex words that readers can draw on to learn about morphemes. For instance, in the 1200 books that we analysed, the suffix *-ance/-ence* only appears in 355 distinct words; yet, 60% (*N* = 213) of these words have stems that do not occur in isolation in these 1200 books. Examples include words like *dominance*, *tolerance*, *benevolence*, *deviance*. The stems *domin*, *toler*, *benevol*, and *devi* cannot stand alone in English, making it unlikely for a child to parse the complex words with these stems into identifiable morphemes. The prefix *sub-* is encountered in 133 different words across these 1200 books, and 58 (44%) of these prefixed words have bound stems that are not used in isolation in English (e.g., *urbia*, *mersion*, *liminal*, *vention* in *suburbia*, *submersion*, *subliminal*, *subvention*). A reader relying solely on orthographic information will struggle to identify the affix morphemes in such words or understand how they contribute to the overall meaning of the word.

Even among the affixes that occur with many distinct stems, a significant portion of these stems are bound and cannot function as standalone words. For example, 26% of the words with the suffix *-y* contain stems that are not used as independent words, and, for the suffixes *-ate* and *-al*, this figure rises to 78% and 51%, respectively. Examples include words like *vasectomy*, *bigamy*, *ignominy*, *eulogy*, *violate*, a*dequate*, *populate*, *imitate*, *initiate*, *equal*, *journal*, *mortal*, *annal*, *abysmal*. Similarly, 33% of the words containing the prefix *re-* have stems that cannot help identify the meaning of this prefix (e.g., *retain*, *revolve*, *receive*, *require*). This issue is even more pronounced for the prefixes *in-* and *im-*, as about 75% of the words containing these prefixes have bound stems (e.g., *inclement*, *indignant*, *insomnia*). The reader may notice that many words contain a specific orthographic pattern (e.g., *re-* or *in-*); however, it is unlikely that they will be able to segment these words into meaningful units or understand how these units combine to convey the overall meaning of the words. To our knowledge, no research has directly examined what children can learn about affixes from morphological constructions such as *inclement* or *insomnia*, where removing the prefix *in-* does not yield a word with a meaning that is clear or directly relatable to the overall meaning of the original construction. This is an important avenue for future research.

Our argument is that some experiences with affixes may be more conducive to morpheme learning than others. This insight provides a foundation for classifying affixes based on psychologically meaningful processes. One classification used in morphology literature is the distinction between ‘neutral’ (e.g., *-ness*, *-able*, *-er*, *-less*) and ‘non-neutral’ suffixes (e.g., *-ity*, *-ion*, *-ic*, *-ance*) [e.g., ref. ^59^]. Research indicates that children aged 13–14 and younger have better knowledge of neutral suffixes and their distributional constraints (e.g., that *-ness* typically attaches to adjectives) compared to non-neutral suffixes^44^. However, no mechanism has been proposed to explain how and why these differences may emerge. Interestingly, suffixes described as neutral in these studies largely align with those that our analysis suggests are used with many distinct stems and are easy to detect, while non-neutral suffixes overlap substantially with those that, in our corpus, are often used with few distinct stems and are more difficult to detect due to their frequent attachment to bound stems. Thus, our findings provide a possible acquisition mechanism that could help connect the abstract linguistic categories ‘neutral’ and ‘non-neutral’ to how human cognition operates.

For some affixes, the situation is further exacerbated by cases of pseudo-affixation, where words appear to contain a specific morpheme but are not actually formed by adding that morpheme to their apparent orthographic stem. In most instances, it is simply a coincidence that the pseudo-affixed words share the same letters and sounds as genuinely affixed words; however, this coincidence can be detrimental to learning, as it may confuse the reader and hinder their understanding of the actual meaning of the affix. To illustrate, out of the 2065 distinct words that actually contain the suffix *-y* (based on MorphoLex tagging), our algorithm was able to identify 1535; however, it also found 505 words that merely *appear* to contain this suffix. Examples include words like *army* (derived from the Latin *armare*, not from the word *arm*), *brandy* (from the Dutch *brandewijn*, not from the word *brand*), *bunny* (originating from the English dialect *bun* meaning ‘squirrel’ or ‘rabbit’, and unrelated to *bun* as in ‘a small cake’ or ‘a hairstyle’), and *forty* (from the Old English *fēowertig*, unrelated to the word *fort*). When we total the words in which *-y* is identifiable (whether correctly or incorrectly, in the case of pseudo-affixed false alarms), we arrive at 2,040 distinct words. However, 25% of these are words like *army* and *brandy*, which happen to end with the letter *y* but were not formed by adding the suffix *-y* to their apparent orthographic stems.

Overall, pseudo-affixation is not widespread among all affixes, which is reassuring as it suggests that it is unlikely to pose a significant challenge for affix learning in general. This finding is also consistent with previous research^54^. However, certain affixes are disproportionately affected. One such example is the prefix *e-*: of the 150 genuinely complex words with this prefix, only 25 can be easily identified as such, while the number of pseudo-affixed words for this prefix reaches 130. To name just a few examples, the word *ebony* appears as though it could be derived from the word *bony*, but it actually originates from the Greek *ebenos*, meaning ‘ebony tree’. Similarly, the word *elate* is unrelated to *late*; it is derived from the Latin *elat*, meaning ‘raised’, from the verb *efferre* ‘to carry out’. The detrimental impact of such words on understanding the function of the prefix *e-* is not trivial: of the 155 words a child might encounter that seem to contain the prefix *e-*, 84% may be misleading or irrelevant.

The implications of this become clear when we consider the acquisition of morphological knowledge through the lens of a general learning framework. In the science of learning, success is believed to depend on the amount of meaningful and relevant data (referred to as ‘signal’) compared to irrelevant and random data (referred to as ‘noise’). Applied to morpheme learning, the signal would represent those genuinely complex words where an affix can be easily detected, while pseudo-affixation would act as background noise. In the best case, this noise is merely distracting, but in some cases, it can significantly hinder learning by diverting readers from the correct meanings of the affixes. Our analyses suggest that, for the majority of affixes, the signal is likely to be too weak to enable learning, while, for some affixes, it may be completely overwhelmed by noise. This means that, beyond a handful of affixes, acquiring morphological knowledge from print will be much more difficult and time-consuming than one might expect based on dictionary-based count data.

This finding may help explain why readers do not appear to engage in rapid morphological analysis until late adolescence^3,46^, despite showing evidence of explicit morphological awareness relatively early in reading acquisition [e.g., refs. ^41–44,74,75^]. The fact that the functions of only a small subset of affixes will be easy to learn from reading experience also helps explain why, in skilled readers, morphological analysis is facilitated for affixes like *-ness* compared to affixes like *-th*^76^. Compared to *-th*, the suffix *-ness* occurs with a much wider range of stems in the 1200 books we analysed and is more easily detectable in the complex words where it appears. The same is true for the comparison between *-ness* and *-ment* and *-ness* and *-al*. We have said above that the development of morphological knowledge in English is thought to be shaped by the information available through print. Our data thus provide insight into why the ability to routinely segment English words into morphemes during online lexical processing takes years to develop and is more readily applied to certain orthographic patterns than others. Research reported here also illustrates how focusing on a single writing system can promote the development of theoretical issues that are relevant to all languages — that readers’ *perception* of morphemes is likely to have a profound impact on their *learning* of morphemes — and demonstrates that the examination of writing systems should be integral to research on morphology acquisition.

These data also prompt us to reframe our thinking about what should be considered as part of a reader’s morphological experience. In the literature, morpheme frequency is typically quantified using dictionary-based counts, where words are considered morphologically complex if they are historically derived from other words [e.g., refs. ^14,17,31,59,68,74^]. Our data indicate that metrics derived from etymologically-based dictionary counts do not accurately reflect lexical experience in the context of affix learning. The fact that many genuinely complex words may not be recognised as such, combined with the presence of morphological false alarms, suggests that any theory of morphological experience that is applicable to real-world behaviour must be based on what readers *can* detect and perceive as morphemes. Readers are unlikely to leverage their experience with words like *sustain*, *submit*, and *subject* to infer the function of the prefix *sub-* if they cannot even identify the prefix *sub-* in these words. Likewise, the average reader may be unable to discern the true signal from the noise and capitalise on relevant cases like *submit* and *subject* while disregarding misleading cases like *subside*. Although encounters with words containing bound stems may still contribute to readers’ awareness of affixes through repeated exposure to certain orthographic patterns (e.g., *sub*), research is yet to determine whether and how this occurs. Moreover, the impact of these encounters on learning likely differs from cases where a word’s morphological structure is clear and can be easily parsed. Thus, our findings suggest that using etymological counts alone as a measure of exposure to morphology is not ecologically valid. Instead, measures of morphological complexity should be based on psychologically realistic learning mechanisms that reflect readers’ real-world language experience.

The formulation of affix learning we have presented implies that improved measures of morpheme exposure should take into account that not all genuinely complex words are equally useful for learning, and that certain aspects of text experience may actively hinder learning (e.g., false alarms). In practice, this would entail that affix frequency counts capture all instances that *appear* to have compositional complexity. This requirement would mean excluding genuinely complex words in which affixes are difficult to recognise, while including unaffixed words whose spelling suggests complexity. Importantly, since these false alarms are likely to have a negative impact on learning, these improved frequency counts would also need to include a penalty associated with these misleading cases.

This approach to quantifying morpheme exposure offers a more accurate representation of readers’ experience with affixes compared to the metrics currently in use. However, even this method does not provide a complete account of morpheme learning, as it does not consider the fact that morphemes contribute to word meaning in a *graded* manner. As we argued earlier, the systematicity with which individual morphemes contribute to word meanings is important because it allows us to understand and create novel forms. For instance, regardless of the specific meaning of the verb *wug*, we instinctively understand that *to rewug* means ‘to wug again’, and that if something is *unwuggable*, it cannot be wugged. Yet, we have also argued that affixes differ in the degree of systematicity with which they convey meaning^13^. As we illustrated in the Introduction, the meaning of the suffix *-ist* is much less consistent than that of the prefix *un-*. For example, the suffix *-ist* appears in words like *typist*, *artist*, *realist*, *tourist*, and *racist*, but it alters the meaning of the stem in a different way in each case. In contrast, the prefix *un-* maintains a consistent meaning across words like *uneven*, *unwise*, *unkind*, and *unabridged*. Laboratory studies have shown that, much like the frequency with which an affix is used with different stems, the consistency of an affix’s meaning plays a crucial role in morpheme learning^58^. Therefore, in addition to considering whether an affix is easily detectable and whether it participates in false alarms, an ecologically valid measure of morpheme learning must also consider the *semantic* consistency of an affix. One way to estimate this is through compositional distributional semantic modelling, which provides a means of representing the meanings of affixes as multidimensional vectors [e.g., refs. ^77,78^]. Once the meanings of affixes are represented in this form, mathematical techniques can be applied to probe different aspects of their meanings. Distributional semantic models require large training datasets, and their application is not always straightforward; nevertheless, they remain a promising avenue for future research.

The implications of our findings also extend meaningfully to questions pertaining to educational practice and instructional design. Popular phonics and morphology guides, such as Blevins^79^, advise focusing instruction on the most common prefixes and suffixes, as these account for the majority of affixes children will encounter in texts. However, our analysis suggests that when affixes are frequent and easily detectable, they should be easy to learn without explicit instruction (but we acknowledge that certain pupils may still benefit from such instruction). Therefore, a more effective approach might be to focus instead on those affixes that appear in a limited number of distinct words and in combination with bound stems. Many of these affixes are of Greek or Latinate origin and attach to stems of the same origin (e.g., prefixes *inter-*, *sub-*, *in-*, *de-*, *e-*, *ex-*, *a-*, *co-*, and suffixes *-ion*, *-al*, *-ate*, *-ance/-ence*, *-ant*, *-ic*, *-ity*, *-ify*). These affixes are often found in complex words that are unlikely to be part of children’s oral vocabulary: examples include words like *indelibly*, *extortionate*, *extemporise*, *palpitate*, *calumniate*, *inaugural*, *bifocal*, *ophthalmic*, *acerbic*, *perpetuity*, *assiduity*, *quantify*. Explicit instruction on the meanings and functions of these morphemes could assist children in interpreting both the individual morphemes and the complex words they form. Yet, some of these affixes are missing from the Blevins list (e.g., prefixes *a-*, *e-*, *ex-*, *co-*, and suffixes *-ory/-ary*, *-ate*, *-ant*, *-ance/-ence*, *-ify*). In the popular books we analysed, these, more difficult, affixes are relatively infrequent but tend to become more common as texts become more advanced. By contrast, they are much more prevalent in expository and scientific texts, which children are increasingly expected to engage with as they progress through the education system [e.g., refs. ^59,80,81^]. Thus, an effective strategy could be to time instruction to coincide with when readers begin to increasingly encounter these morphemes in their real-world reading experience. That said, any changes to the instructional approach should be preceded by a careful examination of how our findings, as well as those related to expository texts, generalise to other types of written materials, such as digital media.

Research assessing the effectiveness of morphology instruction indicates that many instructional programmes fail to bear fruit^82–85^. One reason for this outcome is the significant variability within and across studies regarding what is taught, when it is taught, and to whom [e.g., ref. ^82^]. To reduce this variability, it is essential to close the disconnect between instructional design and children’s everyday reading experience. Our study serves as a critical first step in understanding what children’s exposure to morphology in real-life reading is like and where and when they may require support. For instance, Colenbrander et al.^86^ is one of the few studies that provide information on which affixes were the focus of the intervention. In this study, morphology instruction concentrated on the most common affixes, aiming to help students discover the logic of the English spelling system. Importantly, the trained affixes were also part of England’s Grade 3 Spelling curriculum, meaning that pupils in the intervention group received a double dose of spelling instruction on these affixes compared to their counterparts in the control group. This could be part of the reason why differences between the control and the intervention group were observed in spelling but not in reading measures^86^. Interestingly, the affixes trained in this study largely overlap with those we found to be most common in children’s books and which are expected to be easily detectable. It is therefore possible that the implicit knowledge of these affixes gained through text experience may have reached a threshold beyond which additional instruction offered little to no further benefit. This interpretation aligns with research suggesting that ‘more’ instruction is not always ‘better’ and that there may be a point of diminishing returns^87^. The next logical step would be to examine whether the pattern of results in Colenbrander et al.^86^ would differ for more ‘difficult’ affixes — those that do not occur with many distinct stems, have many false alarms, and are difficult to detect. The optimal dosage of instruction for students with varying abilities also remains a question for future research. That said, it is important to recognise that implementing instructional programmes in morphology presents challenges beyond content and timing. For example, teachers and teaching assistants often find it difficult to deliver these interventions due to the specialised knowledge they require^82,86^. In light of the complexities of implementing morphology instruction at scale, it is imperative that our teaching aligns with the experiences of readers, and that any practical recommendations are developed in collaboration with those responsible for delivering the instruction.

In conclusion, previous research has argued that morpheme learning in English is shaped by experience with print, yet the nature of this experience has remained unclear. We have provided a comprehensive description of the morphological information available in text suitable for children and young people, and have offered the first concrete formulation of how this information may support, or disrupt, affix learning. Our data show that children encounter many morphologically complex words when they read for pleasure. However, only a limited number of affix morphemes are likely to benefit from this exposure in terms of learning, while, for most affixes, exposure is likely to be inadequate to promote learning. This inadequacy arises primarily from the limited repetition of complex words within and across texts, as well as from the fact that the morphological status of many complex words is obscured due to the presence of bound stems and orthographic alterations. Furthermore, most affixes are used with a limited range of distinct stems and require etymological knowledge to be recognised within the complex words in which they occur. Learning can also be hindered by the fact that some words are likely to be segmented into morpheme units even if there is no meaningful relationship between the word and its apparent orthographic stem (e.g., *forty*, *brandy*, *corner*). While the proportion of such words is not large overall, for some affixes, the number of these cases may be substantial enough to have a negative impact on learning. Together, our findings suggest that only about half of the morphologically complex words are likely to contribute meaningfully to children’s experience of individual affixes, ultimately restricting opportunities for affix learning through text. We emphasise that theories of lexical experience and instructional design must reflect the realities of real-world reading experiences and provide insight into what these realities are.

## Methods

All analyses were performed in R, version 4.2.1^88^. Code for all analyses, Supplementary Information, and Supplementary Data 1–4 are available in this project’s repository on the Open Science Framework (https://osf.io/vab95/).

### Data

Our analyses draw on three lexical databases, all of which are publicly available and free to use. The Children and Young People’s Books Lexicon (CYP-LEX)^62^ was used to represent the independent reading experience typical for British children and adolescents aged 7–16. This is the largest database of its kind, providing information on 105,694 distinct English words used in popular children’s books. It is based on a corpus of 1200 texts containing 70,287,217 words and is accessible at 10.17605/OSF.IO/SQU49.

Morphological information based on the etymology of the words in CYP-LEX was extracted from the MorphoLex database^60^ (available at https://github.com/hugomailhot/MorphoLex-en). MorphoLex provides information on the morphological status of 68,624 English words from the English Lexicon Project^89^. The English Lexicon Project builds upon other databases routinely used in psycholinguistic research [e.g., CELEX^14^] and is the largest freely available database that provides psycholinguistic variables and standardised behavioural data for English words that an average native speaker is expected to recognise. We cross-referenced each word in the CYP-LEX database (*N* = 105,694) against MorphoLex, and then extracted the morphological structures of those words that had entries in MorphoLex.

Of all CYP-LEX words, 57,137 (54%) are available in MorphoLex, while 48,557 (46%) are not. The vast majority of the unavailable words are used very infrequently: only 4% (*N* = 1952) appear at least 100 times in the 1200 books in the CYP-LEX corpus, while 93% (*N* = 45,037) are used no more than 50 times across these books. MorphoLex does not include proper names or all possible derivatives of a given stem (e.g., it includes *joyful* and *joyfully*, but not *unjoyfully*), and most of the CYP-LEX words not attested in MorphoLex are absent for these reasons. Among the ‘frequent’ words not attested in MorphoLex, approximately 70% are proper names (e.g., Aidan, Shane, Doreen, McDonald, Lysandra, Bellatrix, Edinburgh, Stockholm). However, the list also includes a small number of multimorphemic words (e.g., *humongous*, *unconvinced*, *gangly*, *shirtless*, *amaurotic*, *legionnaire*, *dismissively*). In contrast, visual inspection of the ‘infrequent’ words not attested in MorphoLex (since no database is available to check the morphological status of these words) suggests that many are morphologically complex (e.g., *fainthearted*, *unghostly*, *unmercifully*, *companionably*, *unclasped*, *unskilful*, *erythroblastic*, *enthrallment*, *voraciousness*). Importantly, because the overwhelming majority of these ‘excluded’ words are used very rarely in children’s books, the 57,137 words for which information is available in MorphoLex can be considered a representative sample. Thus, all analyses were performed on these words.

Finally, the comparison of book language and television language relied on the lexical statistics provided in SUBTLEX-UK^63^ (available at https://psychology.nottingham.ac.uk/subtlex-uk/). This database includes information on the usage of 159,235 distinct words on British television, derived from 201,335,638 words used in programmes broadcast on nine BBC channels (BBC1–BBC4, BBC News, BBC Parliament, BBC HD, CBeebies, CBBC) over three years (2009–2012). In addition to comparing the morphology in CYP-LEX with that attested in the entire SUBTLEX-UK database, we also analysed the differences across CYP-LEX and CBBC, a channel targeting children aged 6–12 years. This age range overlaps with two of the CYP-LEX age bands, 7–9 and 10–12, providing a proxy for the spoken language to which children in this age range are exposed. The CBBC data includes 58,691 distinct words derived from 13,612,278 tokens. Both the CBBC and the complete SUBTLEX-UK databases are substantially larger than any of the subsets of words from the CYP-LEX age bands for which data are available in MorphoLex.

The MorphoLex analysis focused on the most common affixes in the CYP-LEX corpus. An affix was considered common if it appeared in at least 1% of affixed words, analysed separately for prefixes and suffixes. Applying this criterion resulted in a selection of 23 prefixes and 25 suffixes. In general, these sets of affixes were consistent across the age bands, with only a few exceptions. Specifically, the prefix *per-* and the suffix *-n* appeared in 1% of the prefixed or suffixed words in the 7–9 age band but fell below this threshold in the other two age bands. Similarly, the suffixes *-ism* and *-ian* exceeded the 1% threshold only in the 13+ age band. Since these affixes were not consistently present across the age bands, they were excluded from the subsequent analyses.

### The RegEx algorithm

To assess the ease with which the distinct words encountered in children’s books could be segmented into smaller constituents, we designed an algorithm using the Regular Expressions (RegEx) technique. This algorithm evaluated whether a sequence of letters corresponding to each of the 48 most common affixes identified with the MorphoLex analysis (e.g., *er*, *ly*, *un*) was present in CYP-LEX words whose ‘correct’ morphological status (based on the word’s etymology) was available through MorphoLex. Each of these orthographic patterns had to be positioned appropriately (the beginning of the word for the prefixes and the end of the word for the suffixes). If the word did contain the pattern in the correct position, the ‘affix’ was detached, and the algorithm checked whether the remaining letter string (the ‘stem’) was attested in the CYP-LEX database. If it was, the original word was tagged as morphologically complex; if not, the algorithm moved to the next word. For example, the letter sequence *er* appears at the end of the words *teacher*, *corner*, and *infer*. Both *teacher* (genuinely complex) and *corner* (pseudo-affixed) can be segmented into standalone words (*teach*, *corn*) and *er*, and so would qualify as instances of the *-er* suffix based on spelling. The word *infer*, however, would not, as removing *er* leaves *inf*, which is not a valid English word. To process words with multiple affixes, the algorithm was applied recursively until a letter string emerged that could not be split further. For example, the word *soundlessly* contains two suffixes, *-less* and *-ly*. In the first run, the algorithm segmented *soundlessly* into *soundless* (stem) and *-ly* (suffix). In the second run, it split *soundless* into *sound* (stem) and *-less* (suffix).

We enhanced the RegEx algorithm to account for the most common orthographic alterations that occur as a result of derivational affixation. For instance, many English verbs end on a silent *-e* (e.g., $ador\underline{e}$, $rescu\underline{e}$, $creat\underline{e}$). This silent *-e* is often dropped when a suffix beginning with a vowel is added to the word: *adore* + *-able* $\to ado\underline{ra}ble$, *rescue* + *-able* $\to resc\underline{ua}ble$, *create* + *-or* $\to crea\underline{to}r$. Another common orthographic alteration is consonant doubling: *sun* + *-y* $\to su\underline{nn}y$, *run* + *-er* $\to ru\underline{nn}er$, *admit* + *-ance* $\to admi\underline{tt}ance$. We aimed to apply rules that children might be aware of; therefore, we made sure that the selected rules were taught as part of the English National Curriculum for English spelling^90^ (see Supplementary Information for a full list of rules that we used).

To determine how many genuinely multimorphemic words could be identified as such by a reader familiar with the most common orthographic rules, we applied the RegEx algorithm to the subset of words marked as morphologically complex in MorphoLex. Initially, we conducted this analysis separately for each age band. However, because the pattern of results was nearly identical across the age bands, we subsequently applied the RegEx algorithm to all CYP-LEX words identified as morphologically complex in MorphoLex, without differentiating by age band, to simplify the analysis and reporting of results. The CYP-LEX database includes 105,694 distinct words. Information from MorphoLex was available for 54% of these words (*N* = 57,137), and of these, 51% (*N* = 29,244) were tagged as morphologically complex. 17% (*N* = 5029) of the complex words were compounds that did not include any affixes, leaving 24,215 words for analysis. Of these, 8473 included at least one prefix, and 19,635 included at least one suffix (note that some words included both prefixes and suffixes, which is why these totals exceed 24,215). The RegEx algorithm consisted of two components: one designed to detect prefixes and the other to detect suffixes. The prefix component was applied to words with at least one prefix, and the suffix component was applied to words with at least one suffix. The algorithm identified prefixes in 4141 words (49% of all words with prefixes) and suffixes in 13,643 words (69% of all words with suffixes).

To estimate the number of words likely to be classified as prefixed or suffixed based on their spelling but lacking actual prefixes or suffixes (i.e., potential false alarms), we applied the RegEx algorithm to CYP-LEX words tagged in MorphoLex as having no affixes. This analysis included 48,644 words without prefixes and 37,502 words without suffixes. Of these, the RegEx algorithm classified 1654 (3%) as prefixed and 1951 (5%) as suffixed. These classifications occurred because removing an orthographic pattern corresponding to one of the 23 prefixes or 25 suffixes produced a standalone word in the CYP-LEX corpus (e.g., removing the *er* from *corner* leaves the word *corn*).

For each prefix and suffix, Supplementary Data 2 and 3 list the prefixed (Supplementary Data 2) and the suffixed (Supplementary Data 3) words that were detected by the RegEx algorithm, based on data derived from all 1200 books. Supplementary Data 4 provide information on the total number of words with a given affix (i.e., type frequency of the affix), the number of affixed words that were detected by our algorithm, and the number of words that were incorrectly identified as containing this affix based on their spellings (i.e., pseudo-affixed words). As with any analysis of natural language, the RegEx algorithm has limitations. The algorithm flags words as potential false alarms if removing an affix-like pattern results in a letter string found in CYP-LEX. However, CYP-LEX includes proper names, interjections, jargon, and words coined by authors within specific books that may be unfamiliar to readers outside those contexts. These words are unlikely to be treated as potential stems by every reader. For instance, the database includes interjections like *aaargh*, which the algorithm classifies as a potential false alarm for the prefix *a-* because *aargh* is also attested in CYP-LEX. There is no automated way to detect such cases, but, while they do occur, they are unlikely to be numerous. Nonetheless, this example demonstrates that, since the RegEx algorithm relies solely on spelling and does not consider word meanings, in some cases its approach to complex-looking words may differ from that of a human reader. For this reason, we recommend focusing not on the absolute values produced by the algorithm (e.g., the exact number of words in which an affix could or could not be detected) but on the general trends that the output reveals regarding the visibility of affixes in the words we analysed.

## Supplementary information

Supplementary Information

Supplementary Data

## Data Availability

All data analysed in this article are publicly available. Links to the data are provided in the Methods section of this article and in this project’s repository on the Open Science Framework at https://osf.io/vab95/.

## Code availability

The code for all analyses is available in this project’s repository on the Open Science Framework at https://osf.io/vab95/.
