# Supplementary material for: Morphology in children’s books, and what it means for learning
Source: NPJ Sci Learn. 2025 May 5;10:22. doi: 10.1038/s41539-025-00313-6 (PMC12053585; doi:10.1038/s41539-025-00313-6)
Supplement: Supplementary file 1 — Supplementary Information [file 41539_2025_313_MOESM1_ESM.pdf]

Morphology in children's books, and what it means for learning

Maria Korochkina & Kathleen Rastle

Supplementary Information: *Orthographic rules incorporated in the RegEx algorithm*

1. Silent *-e* deletion

The *-e* at the end of the stem is dropped before any suffix beginning with a vowel letter: e.g., *create* + *-or* → *creator*, *make* + *-er* → *maker, cube* + *-ic* → *cubic, argue + -ment* → *argument*, *diverse* + *-ity* → *diversity*.

2. Consonant doubling

The last consonant letter of the stem may be doubled when the suffix that is being added starts with a vowel: e.g., *sun* + *-y* → *sunny*, *run* + *-y* → *runny*.

3. *-y* changes to *-i*

The *-y* at the end of the stem is changed to *-i* when a suffix starting on a consonant is added and the stem has more than one syllable: e.g., *beauty* + *-ful* → *beautiful*, *merry* + *-ment* → *merriment*, *plenty* + *-ful* → *plentiful*, *happy* + *-ly* → *happily*.

4. For the suffix *-ous:*

   a. *-our-* in the stem may change to *-or-*: e.g., *humour* + *-ous* → *humorous*, *glamour* + *-ous* → *glamorous*, *vigour* + *-ous* → *vigorous*.
   b. *-e* at the end of the stem changes to *-i* if the stem has more than 2 letters: e.g., *grace* + *-ous* → *gracious*.

5. For the suffix *-ion*:

   a. *-t* at the end of the stem may change to *-ss*: *permit* + *-ion* → *permission*.
   b. *-d* at the end of the stem may change to *-t*: *attend* + *-ion* → *attention*.
   c. *-d(e)* at the end of the stem may change to *-s*: *expand* + *-ion* → *expansion*, *include* + *-ion* → *inclusion*.

6. *-ou-* changes to *-u-*

-ou- in the stem may change to *-u-* when a suffix is added: e.g., *abound* + *-ance* → *abundance*.

7. For the prefix *in-*:
   a. *in-* becomes *il-* if the stem starts with an *-l*: e.g., *in-* + *legal* → *illegal*.
   b. *in-* becomes *im-* if the stem starts with an *-m*: e.g., *in-* + *mature* → *immature*.
   c. *in-* becomes *ir-* if the stem starts with an *-r*: e.g., *in-* + *regular* → *irregular*.

Supplementary Data: *Supplementary Data 1−4*

Supplementary Data 1. An Excel (.xlsx) file listing the type and token frequency of the 48 most common affixes in the CYP-LEX corpus.

Supplementary Data 2. An Excel (.xlsx) file listing *prefixed* words identified in the CYP-LEX corpus based on the MorphoLex analysis, including their lexical statistics and whether they were detected by the RegEx algorithm.

Supplementary Data 3. An Excel (.xlsx) file listing *suffixed* words identified in the CYP-LEX corpus based on the MorphoLex analysis, including their lexical statistics and whether they were detected by the RegEx algorithm.

Supplementary Data 4. An Excel (.xlsx) file detailing the type frequency of the 48 most common affixes in the CYP-LEX corpus (according to the MorphoLex analysis), along with the number of distinct words with each affix that were detected by the RegEx algorithm and the number of distinct words incorrectly identified as containing the affix based on their spelling (i.e., false alarms).

Please note that *Supplementary Information* and *Supplementary Data 1–4* are also available in this project's repository on the Open Science Framework: https://osf.io/vab95/.
